# The role of neutrophil elastase in aortic valve calcification

**DOI:** 10.1186/s12967-022-03363-1

**Published:** 2022-04-09

**Authors:** Yan Liu, Peng Jiang, Liqin An, Mengying Zhu, Jin Li, Yue Wang, Qin Huang, Yi Xiang, Xiaorong Li, Qiong Shi, Yaguang Weng

**Affiliations:** The Department of Laboratory Medicine, M.O.E. Key Laboratory of Laboratory Medical Diagnostics, Chongqing Medical University, Chongqiong, China

**Keywords:** Calcific aortic valve diease, Neutrophil elastase, Valve interstitial cells, Osteogenic differentiation, Inflammation

## Abstract

**Background:**

Calcific aortic valve disease (CAVD) is the most commonly valvular disease in the western countries initiated by inflammation and abnormal calcium deposition. Currently, there is no clinical drug for CAVD. Neutrophil elastase (NE) plays a causal role in inflammation and participates actively in cardiovascular diseases. However, the effect of NE on valve calcification remains unclear. So we next explore whether it is involved in valve calcification and the molecular mechanisms involved.

**Methods:**

NE expression and activity in calcific aortic valve stenosis (CAVD) patients (n = 58) and healthy patients (n = 30) were measured by enzyme-linked immunosorbent assay (ELISA), western blot and immunohistochemistry (IHC). Porcine aortic valve interstitial cells (pVICs) were isolated and used in vitro expriments. The effects of NE on pVICs inflammation, apoptosis and calcification were detected by TUNEL assay, MTT assay, reverse transcription polymerase chain reaction (RT-PCR) and western blot. The effects of NE knockdown and NE activity inhibitor Alvelestat on pVICs inflammation, apoptosis and calcification under osteogenic medium induction were also detected by RT-PCR, western blot, alkaline phosphatase staining and alizarin red staining. Changes of Intracellular signaling pathways after NE treatment were measured by western blot. Apolipoprotein E^−^/^−^ (APOE^−^/^−^) mice were employed in this study to establish the important role of Alvelestat in valve calcification. HE was used to detected the thickness of valve. IHC was used to detected the NE and α-SMA expression in APOE^−^/^−^ mice. Echocardiography was employed to assess the heat function of APOE^−^/^−^ mice.

**Results:**

The level and activity of NE were evaluated in patients with CAVD and calcified valve tissues. NE promoted inflammation, apoptosis and phenotype transition in pVICs in the presence or absence of osteogenic medium. Under osteogenic medium induction, NE silencing or NE inhibitor Alvelestat both suppressed the osteogenic differentiation of pVICs. Mechanically, NE played its role in promoting osteogenic differentiation of pVICs by activating the NF-κB and AKT signaling pathway. Alvelestat alleviated valve thickening and decreased the expression of NE and α-SMA in western diet-induced APOE^−^/^−^ mice. Alvelestat also reduced NE activity and partially improved the heart function of APOE^−^/^−^mice.

**Conclusions:**

Collectively, NE is highly involved in the pathogenesis of valve calcification. Targeting NE such as Alvelestat may be a potential treatment for CAVD.

**Supplementary Information:**

The online version contains supplementary material available at 10.1186/s12967-022-03363-1.

## Background

Calcified aortic valve disease (CAVD) is a heart valvular disease commonly occurring in the elderly over 65 years old [1]. The pathological process of CAVD involves inflammation, matrix remodeling, thickening and calcification of valve leaflets, and more serious, it may progress to calcified aortic valve stenosis (CAVD) [2]. Currently, there is no clinically effective treatments for CAVD except aortic valve replacement surgery [3]. Therefore, therapeutic targets and drugs for CAVD need to be further developed and explored.

NE (Neutrophil elastase) is a serine protease mainly existed in neutrophils azurophilic granules [4], which is involved in a variety of physiological processes like formation of neutrophil extracellular trap and degradation of extracellular matrix and proteins [5, 6]. Due to it can rapidly released from neutrophils in response to inflammatory signals, NE is often thought to be a sign of inflammation and significantly contributes to some inflammatory diseases such as bronchiectasis [7] and chronic obstructive pulmonary disease [8]. Besides, NE also actively participates in cardiovascular disease. In patients with coronary heart disease, especially in patients with unstable angina and acute myocardial infarction, the level of NE is significantly increased, which is considered to be a predictor of cardiovascular events [9]. NE increases myocardial damage by inducing excessive inflammation [10]. It has been reported that NE was highly expressed in atherosclerotic plaques and its inhibitor effectively remitted the instability of atherosclerotic plaques, suggesting that it plays an important role in atherosclerosis [11]. Meanwhile, we previously demonstrated that 45KD GRN (Granulin), a small fragment of PGRN (Progranulin), significantly increased in calcified valves and had the effect of promoting inflammation and calcification [12]. It is well known that NE can degrade PGRN into small fragments of peptides [13]. Considering that NE is closely related to inflammation and cardiovascular disease, we speculated that the emergence of 45KD GRN may be related to NE and NE might be involved in the pathological process of CAVD.

Here, we detected the level of NE in CAVD patients and further studied the role of NE in aortic valve calcification for the first time,with the aim of identifying new therapeutic targets and drugs for the treatment of CAVD.

## Materials and methods

### Human aortic valve tissues and serum

Normal human AV tissue was collected from a patient with aortic dissection, and calcified AV tissues were collected from three patients with calcific aortic valve disease undergoing aortic valve replacement. All the valve tissues were rapidly cut in two parts. One part was fixed in 4% paraformaldehyde, the other was frozen and stored in liquid nitrogen.

Serum was collected from 58 patients diagnosed with aortic valve stenosis. As controls, normal serum were obtained from 30 healthy people (Additional file 1: Table S1). Serum with hemolysis or blood lipid was excluded and stored at – 80 ℃ until assays were performed.

This study was approved by the Ethics Committee of Chongqing Medical University. Informed consent was obtained from all the patients and their family members.

### Animals

All animal procedures were approved by the Ethics Committee of Chongqing Medical University. The male Apoe^−^/^−^ mice were obtained from the Animal Experiment Center of Chongqing Medical University. Six to 8 weeks mice were divided into four groups: normal diet (ND), normal diet with Alvelestat (ND + Alv), western diet (WD), western diet with Alvelestat (WD + Alv). WD group and WD + Alv group were fed western diet containing 40% fat for 16 weeks (XT108C, Jiangsu Xietong Pharmaceutical Bio-engineering Co., Ltd.). A dose of 3 mg/kg Alvelestat was used in this study to pharmacologically inhibit the NE activity in mice once a week based on the previous studies [14].

### Echocardiography

Echocardiography was conducted on mice using a Vinno 6 ultrasound system (VINNO, Suzhou, China) in this study. Mice were anesthetized by induction for 5 min with 5% isoflurane and maintained at 1.5% isoflurane with an oxygen flow rate of 2.0 L/min. The heart is imaged in a two-dimensional parasternal long axis view and an M-echocardiogram of the middle chamber is recorded at the myocardial level. Cusp separation and ejection fraction were obtained from the M-mode image. Doppler measures of blood velocity were all made with the intercept angle 60° between the targeted vessel and ultrasound beam.

### Cell isolation and culture

The porcine aortic valve interstitial cells (pVICs) were isolated and cultured as previously reported [12]. In brief, the pVICs were removed in the sterile environment. In the biological safety cabinet, the surfaces of the valves were wiped repeatedly with sterile cotton swabs to remove the valve endothelial cells. Then valves were cut into small pieces of 2 × 2 mm and subjected to an 8-h collagenase digestion at 37 ℃. Then, pVICs were cultured in Media 199 (Hyclone, USA) supplemented with 10% fetal bovine serum (Cellmax, USA) and 1% penicillin/streptomycin (C0222, Beyotime Biotechnology). pVICs from passages 3–7 were used to following experiment.

To induce calcification, pVICs were cultured in osteogenic medium: M199 supplemented with 10% FBS, 1% penicillin/streptomycin, 10 mmol/L β-glycerin glyphosate (ST637, Beyotime Biotechnology), 50 µg/mL ascorbic acid (ST1434, Beyotime Biotechnology) and 100 nmol/L dexamethasone (ST1258, Beyotime Biotechnology). pVICs were treated with 1 µg/mL active human neutrophil elastase protein (ab91099, Abcam) for 12, 24, 48 h to assess its effect on pVICs apoptosis, phenotypic transition and inflammation. To further verify the effect of NE on pVICs calcification, pVICs were cultured in osteogenic medium with NE, 40 nmol/L Alvelestat (T3107, Topscience) and both. To further illustrate the different functions of PGRN and 45KD GRN on valve calcification, pVICs were added with 800 ng/mL recombinant PGRN protein or transfected with 45KD GRN virus or contrls virus in the presence of OM. In order to further verify the degradation effect of NE on PGRN, HEK293 cells were cultured in DMEM (Hyclone, USA) supplemented with 10% fetal bovine serum and 1% penicillin/streptomycin. Cells were added with 800 ng/mL recombinant PGRN protein with or without NE and Alvelestat. For the study of intracellular signaling pathways, pVICs were treated with HNE in the absence or presence of osteogenic medium for 24 h. The later signaling pathway inhibitor were added 1 h before stimulation with HNE: the NF-ĸB inhibitor BAY 11-7082 (Cat. No. 196870, Calbiochem), and the Akt inhibitor LY294002 (S1105, Selleckchem).

### ELISA

Detection of NE levels in serum was performed using ELISA according to the manufacturer’s standard protocol (DY008, R&D Systems).

### Measurement of NE activity

NE activity in serum and supernatant were measured according to the procedure used by Virca, G.D. et al [15]. Briefly, the serum and supernatant were transferred into 96-well plates with 500 µM substrate solution N-methoxysuccinyl-Ala-Ala-pro-Val-p-nitroanilide(M4765, Sigma-Aldrich) in 20 mM Tris–HCl, pH 7.5, with the addition of 10 µg/mL human serum albumin (bs-0292P, Bioss) and incubated for 24 h at room temperature. The absorbance of N-methoxysuccinyl-Ala-Ala-pro-Val-p-nitroanilide cleaved by NE was measured at 405 nm.

### Real-time polymerase chain reaction

Total RNA was extracted using RNA-quick purification kit (RN001, Esunbio) according to the manufacturer’ s recommendations. Reverse transcriptase reactions were performed using PrimeScriptTM RT Master Mix (RR036B, Takara). qRT-PCR was performed using 2X SYBR Green Fast qPCR Mix (RK02001, Biomarker) on a CFX Connect Real-Time PCR Detection System (Bio-Rad Laboratories, Hercules, CA, USA). All primer sequences are shown in Additional file 1: Table S2. Data were normalized by GAPDH expression and expressed as fold change relative to controls. All PCRs were performed at least in triplicate for each experimental condition.

### Immunohistochemistry

All the AV tissues were dehydrated, embedded in paraffin, and cut in 5-µm-thick sections. The sections were deparaffinized with xylene and hydrated with gradient alcohol. For haematoxylin and eosin stain, sections were stained with hematoxylin (G1120, Solarbio) for 1 min and then with eosin for 1 min. For Alizarin red S staining, sections were stained with Alizarin Red S staining working solution (G3280, Solarbio) for 5 min and hematoxylin for 1 min. For immunohistochemical staining, after antigen retrieval with sodium citrate, sections were treated with 3% hydrogen peroxide for 10 min to block endogenous peroxidase and blocked by 5% normal goat serum (SP-9000, ZSGB-BIO) for 15 min, sections were incubated with primary antibodies against OPN and NE overnight at 4 ℃.Then sections were further incubated with biotin-labeled goat anti-rabbit IgG for 15 min and HRP-labeled Streptomyces ovoalbumin working solution for 15 min. The signal was revealed by using a DAB Substrate Kit (ZLI-9017, ZSGB-BIO).

### Immunofluorescence staining

Cultured pVICs were fixed in 4% paraformaldehyde and permeabilized by 0.5% Triton X-100(T8200, Solarbio). After blocked by 5% bovine serum albumin at room temperature for 30 min, cells were incubated with primary antibody overnight at 4 ℃ followed by AlexaFluor 647-conjugated anti-rabbit secondary antibodies (bs-0369 M, Bioss) for 2 h at room temperature in dark. Nuclei were stained with DAPI (C0065, Solarbio) for 10 min. Images were captured by Fluoview FV1000 laser scanning confocal system (Olympus, Tokyo, Japan).

### NE knockdown by siRNA

A pool of three target-specific siRNAs was used to silence NE expression. The siRNAs targeting porcine NE transcript were designed and synthesized from RiboBio. A negative control (NC) siRNA was used as a scramble siRNA, targeting no known gene. Targeted sequences are as follows: siNE#1: CCGACGCTATGGTGATAAT; siNE#2: GCAGCATCATGCAGCATCT; siNE#3: GCAGCAGCTCAATGTGACT. pVICs reached at 70–80% consistency were transfected with 5 nM NE or NC siRNAs using Lipofectamine 2000 Transfection Reagent (Cat. No. 11668500; Invitrogen; Thermo Fisher Scientific, Inc.). Cell lysates were collected and examined for NE mRNA expression 24 h and protein expression 48 h after transfection.

### Western blot analysis

Total protein were prepared by lysing the cells in RIPA buffer supplemented with protease inhibitors and phosSTOP phosphatase inhibitors (Cat. No. 56-25-7, Roche). The protein samples (20 µg) were separated by electrophoresis on a 10% SDS–polyacrylamide gel and transferred to a polyvinylidene fluoride membrane (C3117, Millipore). After blocking with 5% skimmed milk powder at 37 ℃ for 2 h, use the primary antibody overnight at 4 ℃ to detect the protein of interest (Additional file 1: Table S3). After washed three times in TBST, the membranes were incubated with corresponding secondary antibodies conjugated with horseradish peroxidase (ZB-2305, ZSGB-BIO) at dilution of 1:5000 for 1 h at room temperature. Finally, the membranes were visualized by enhanced chemiluminescence (4AW011-200B, 4A BIOTECH) and calculated the gray value of each band by using Image Lab software. The internal control GAPDH was used as control, and the ratios of the gray value of the target protein bands to the gray value of the corresponding internal control bands were used as the expression level of the target protein.

### TUNEL assay

pVICs were seeded into the sterile coverslip (BS-14-RC, Biosharp) at a density of 1 × 10^3^ cells/well. After the cells are completely adherent, 1 µg/mL NE was added into the cells for 24 h. Then, apoptotic cells were detected using TUNEL assay kit (E-CK-A321, Elabscience) according to the manufacturer's standard protocol.

### MTT assay

pVICs in the logarithmic growth phase were seeded into 96-well plates at a density of 5 × 10^3^ cells/well with three replicate wells. After 8 h incubation, cells were treated with 1 µg/mL NE for 24 h. The medium was removed and 10% MTT solution (5 g/L, M8180, Solarbio) was added into the plates. After 4 h incubation, the supernatant was discarded and 100ul DMSO (D8371, Solarbio) was added to each well. The absorbance at 490 nm was valued by the microplate reader.

### Alkaline phosphatase and alizarin red staining

pVICs were fixed in 4% paraformaldehyde for 20 min and washed in PBS for 5 min. Then, cells were incubated with 200ul alkaline phosphatase solution (C3206, Beyotime Biotechnology) or alizarin red solution (130-22-3, Solarbio) for 30 min. Cells were washed in distilled water for 5 min and then observed using a Leica DM IL LED microscope.

### Statistical analysis

All experiments were performed in triplicate per treatment condition. Data are presented as means ± SD. Comparisons between values of different groups were analyzed by one-way ANOVA or Student t-test for multigroups or two groups individually. GraphPad Prism software (Version 9.0) was used for statistical analysis. *P* < 0.05 was considered statistically significant.

## Results

### The levels and activity of NE are elevated in patients with CAVD

**Fig. 1:**
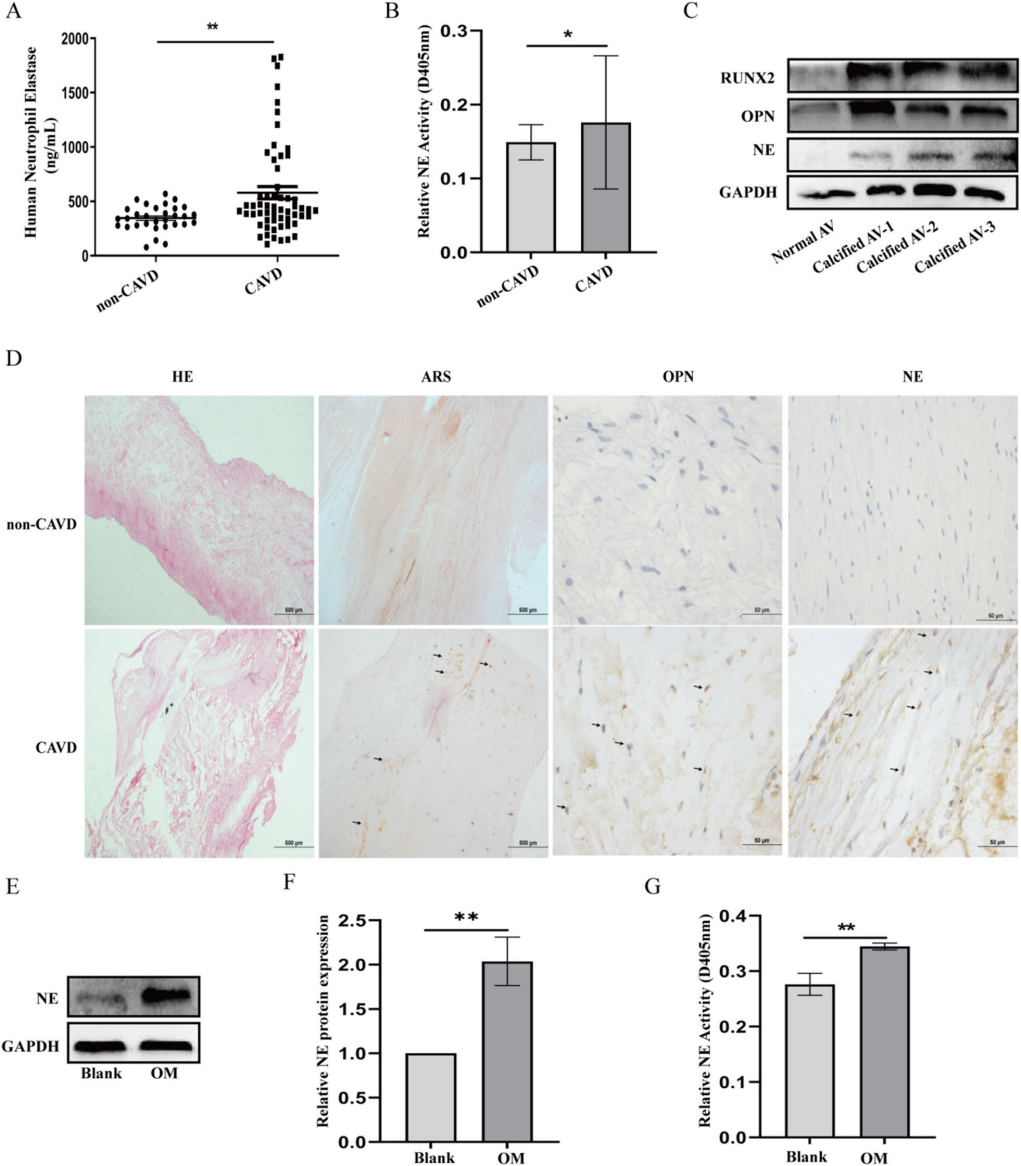
The expression and activity of NE in the serum and calcified aortic valve from patients with CAVD. **A** Detection of the circulating levels of NE in patients with CAVD and non-CAVD by ELISA. **B** NE activity in patients with CAVD and non-CAVD was measured by N-methoxysuccinyl-Ala-Ala-pro-Val-p-nitroanilide. For non-CAVD patients, n = 30. For CAVD patients, n = 58. *P < 0.05, **P < 0.01 *vs* non-CAVD group. **C** Western blot analysis of RUNX2, OPN and NE protein expression in the normal AV and calcified AV. *P < 0.05, **P < 0.01 vs normal AV. **D** Representative micrographs of aortic valve sections from patients with CAVD and non-CAVD for HE, ARS, OPN and NE. The black arrow indicates the positive area. Scar bar = 500 µm, Scar bar = 50 µm. **E**, **F** WB analysis of NE expression and **G** detection of NE activity in pVICs cultured in osteogenic medium for 24 h. **P < 0.01 vs Blank group. The measurement data are expressed as the mean ± SD. All experiments were independently repeated three times

To investigate whether NE plays an important role in valve calcification, we detected the levels and activity of NE in patients with CAVD. As shown in Fig. 1A and B, the level and activity of NE in patients with CAVD were significantly higher than in non-CAVD group. Moreover, immunohistochemical analysis and Western blot results further showed that NE expression was increased in calcified valve tissues contrast with normal valve tissue (Fig. 1C, D). Besides, after pVICs were treated with osteogenic medium for 24 h, the expression and activity of NE were significantly increased (Fig. 1E–G).

### NE promotes apoptosis, inflammation and phenoical transformation in pVICs

**Fig. 2:**
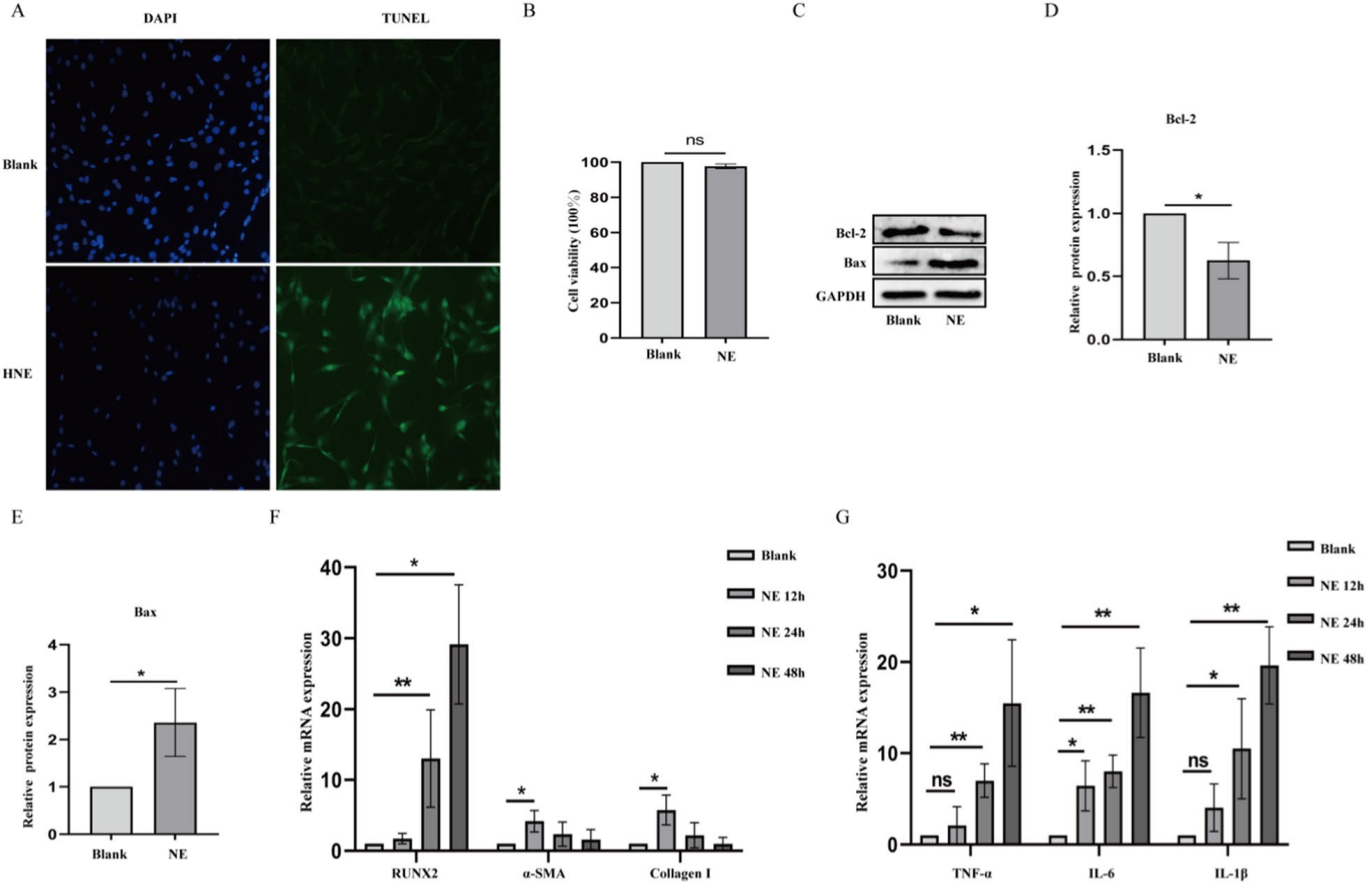
The effect of NE on apoptosis, inflammation and activation of pVICs. **A** TUNEL assay was used to value apoptosis of pVICs treated with HNE for 24 h. Scar bar = 100 µm. **B** MTT assay detection of proliferation in pVICs treated with NE for 24 h. **C**–**E** Western blot analysis of Bcl-2 and Bax protein expression. **F**, **G** RT-qPCR detection of RUNX2, α-SMA, Collagen I, TNF-α, IL-6 and IL-1β mRNA expression in pVICs treated with NE for 12, 24 and 48 h. *P < 0.05, **P < 0.01 vs Blank group. NS meant no significant difference. The measurement data are expressed as the mean ± SD. All experiments were independently repeated three times

We then further established the role of NE on apoptosis and inflammation in pVICs. Immunofluorescence results presented that fluorescence intensity was enhanced after HNE treatment, suggesting that NE can enter into cells to function (Additional file 1: Fig. S2). The result of TUNEL assay showed NE treatment induced more apoptosis of pVICs (Fig. 2A) while no obvious effect on cell proliferation (Fig. 2B). Meanwhile, NE promoted the increase of bax and the decrease of bcl-2 in pVICs (Fig. 2C–E). We then examined the role of NE in phenotypic transformation and inflammation in pVICs at different time points. NE increased α-SMA and Collagen I mRNA expression at 12 h and elevated RUNX2 mRNA expression from 24 to 48 h of treatment (Fig. 2F). In addition, NE increased the expression of inflammatory factors TNF-α, IL-6 and IL-1β (Fig. 2G).

### NE promotes pVICs calcification induced by osteogenic medium

**Fig. 3:**
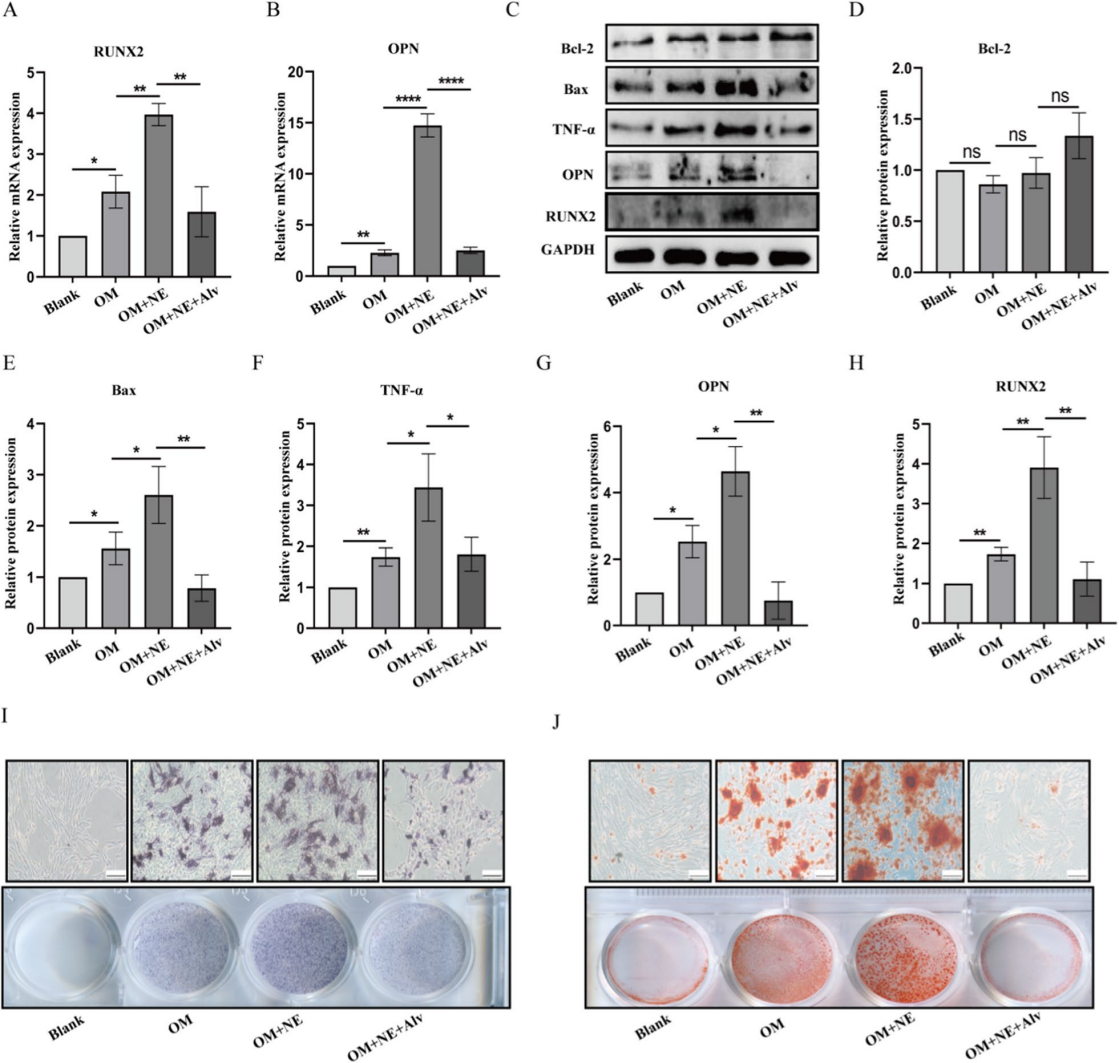
The effect of NE on pAVIC calcification induced by osteogenic medium. pVICs were cultured in osteogenic medium combined with NE, Alvelestat or both for 24 h. **A**, **B** RT-qPCR detection of RUNX2 and OPN. **C**–**H** Western blot analysis of bcl-2, bax, TNF-α, RUNX2 and OPN. **I** The degree of osteoblastic differentiation was measured by ALP staining. **J** Calcium deposit was measured by Alizarin Red Staining. Scar bar = 100 µm. *P < 0.05, **P < 0.01, ****P < 0.0001 vs corresponding control groups. NS meant no significant difference. The measurement data are expressed as the mean ± SD. All experiments were independently repeated three times

As shown in Fig. 3A–H, NE induced a significant increase of RUNX2 and OPN at both mRNA and protein levels under the induction of osteogenic medium. Besides, NE also stimulated the expression of TNF-α and bax, although no elevation of bcl-2. For confirmation, pVICs were treated with NE in combination with its inhibitor Alvelestat for 24 h in osteogenic medium. The mRNA and protein results indicated that Alvelestat prevented NE from promoting the increase of RUNX2, OPN, TNF-α and bax. ALP staining and Alizarin Red Staining results showed that the number of ALP positive cells and calcium deposition were markedly increased after 7 or 14 days of incubation in osteogenic medium in the presence of NE, compared with OM group. However, Alvelestat still reversed this effect (Fig. 3I, J).

### Specific inhibition of NE activity attenuates OM-induced pVICs calcification

**Fig. 4:**
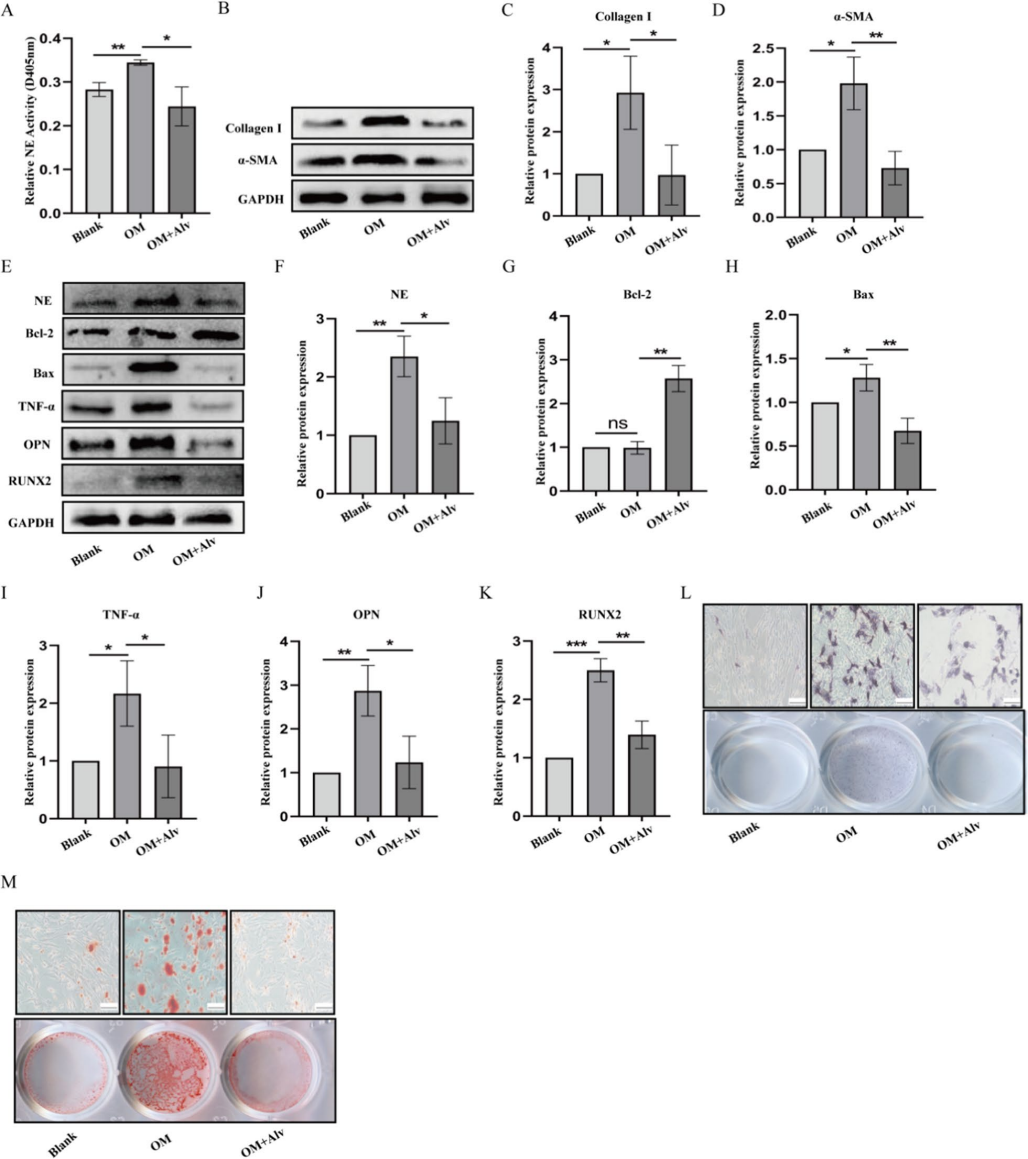
Effects of NE inhibtor Alvelestat on pVICs osteogenic differentiation induced by OM. pVICs were treated with 40 nmol/L Alvelestat for 12 h or 24 h in osteogenic medium. **A** Dection of NE activity by N-methoxysuccinyl-Ala-Ala-pro-Val-p-nitroanilide. **B**–**D** Western blot analysis of fibrosis markers α-SMA and Collagen I in 12 h. **E**–**K** Western blot analysis of NE, TNF-α, osteogenic markers (RUNX2, OPN) and apoptosis proteins(bcl-2, bax) in 24 h. **L**, **M** ALP staining and Alizarin Red Staining of pVICs cultured in osteogenic medium for 7 or 14 days. *P < 0.05, **P < 0.01, ***P < 0.001 vs corresponding control groups. NS meant no significant difference. The measurement data are expressed as the mean ± SD. All experiments were independently repeated three times

To verify whether inhibition of NE activity affects pVICs calcification, pVICs were treated with 40 nmol/L Alvelestat to observe its effect on calcification, inflammation and apoptosis related indicators. As shown in Fig. 4A, Alvelestat reduced the increase of NE activity induced by OM. Also, Alvelestat inhibited early fibrosis index α-SMA and Collagen I at 24 h (Fig. 4B–D) and inhibited calcification indicators RUNX2 and OPN, inflammatory factor TNF-α, apoptosis protein bax as well as NE at 48 h (Fig. 4E–K). Furthermore, Alvelestat reduced ALP staining and calcium deposition (Fig. 4L, M).

### NE knockdown ameliorates pVICs calcification

**Fig. 5:**
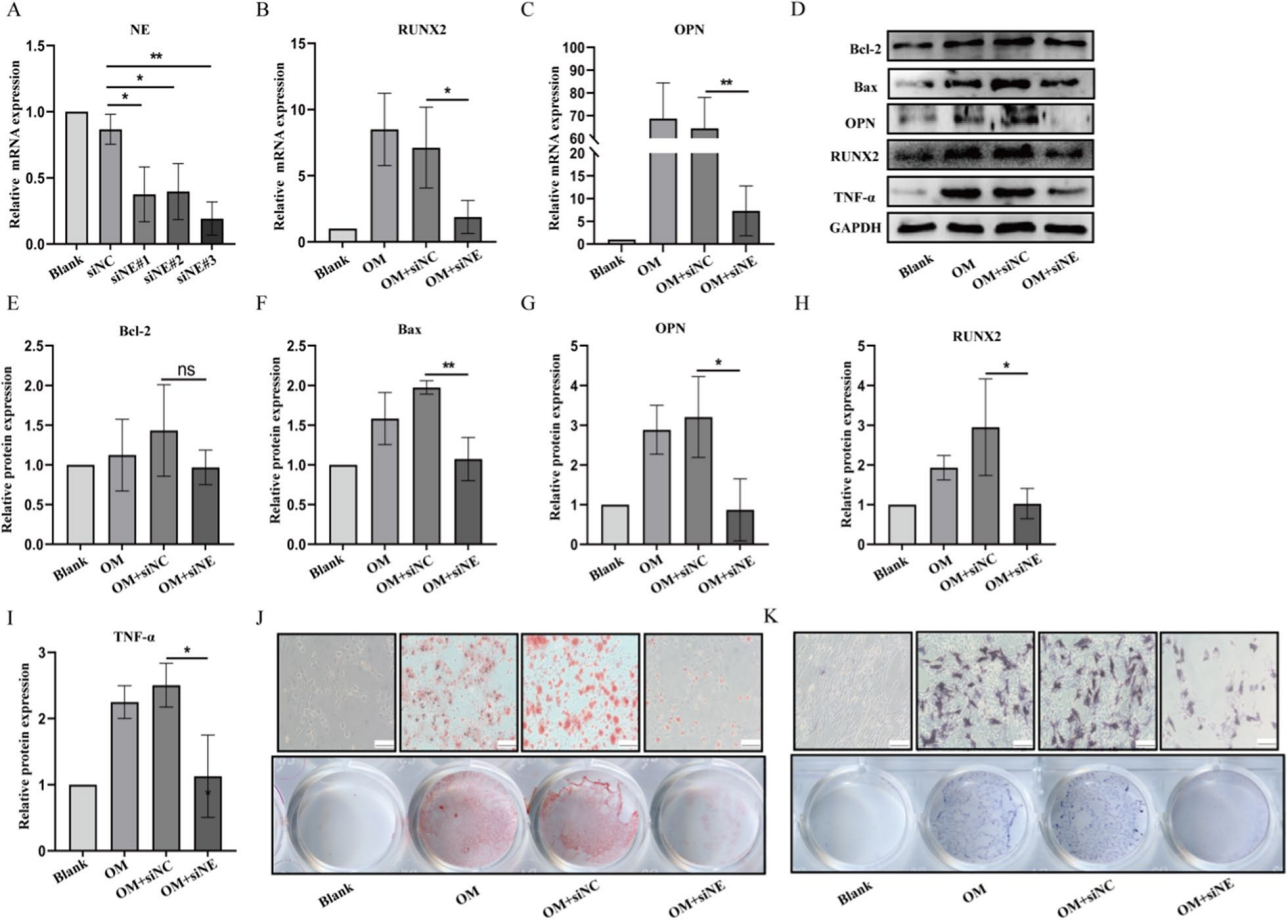
Effects of silencing NE on pVICs calcification. pVICs were transfected with 5 nM siRNA targeted NE or negative control for 24 h. **A** RT-qPCR detection of siRNA targeted NE knockdown efficiency. **B**, **C** RT-qPCR detection of mRNA expression of RUNX2 and OPN in each groups. **D**–**I** Western blot analysis of TNF-α, osteogenic markers (RUNX2, OPN) and apoptosis proteins (bcl-2, bax) in each groups. **J** Calcium deposition was measured by Alizarin red staining in pVICs cultured in osteogenic medium for 14 days. **K** Early osteogenic differentiation was measured by ALP staining in pVICs cultured in osteogenic medium for 7 days. *P < 0.05, **P < 0.01 vs corresponding control groups. NS meant no significant difference. The measurement data are expressed as the mean ± SD. All experiments were independently repeated three times

We have previously demonstrated that NE expression was elevated during valve calcification. Next, we transfected Ne-targeted siRNA into pVICs to verify the effect of NE knockdown on valve calcification. The NE knockdown efficiency was validated by markedly diminished NE mRNA level in pVICs (Fig. 5A). With NE knockdown in pAVICs, we found reduced mRNA and protein levels of RUNX2 and OPN. In line with this, NE silencing also inhibited TNF-α and bax (Fig. 5B–I). In addition, both Alizarin red and ALP staining results displayed that NE deficiency decreased the osteogenic differentiation and calcium deposition (Fig. 5J, K).

### Up-regulation of 45KD GRN is due to the cleavage of NE

**Fig. 6:**
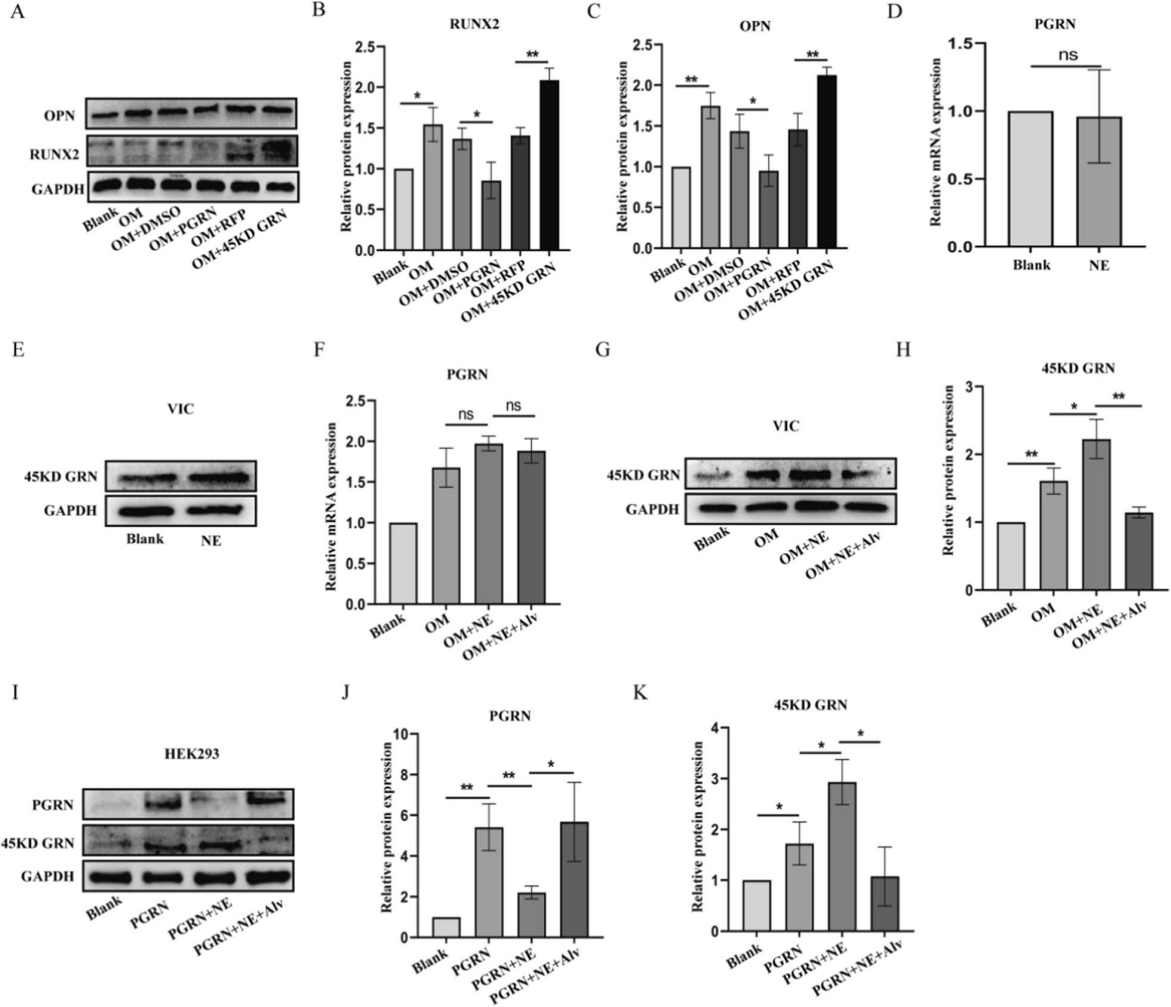
The effect of NE on the degradation of PGRN. **A**–**C** pVICs were added with 800 ng/mL human recombinant PGRN protein or dimethyl sulfoxide (DMSO) and were transfected with red fluorescent virus (Ad-RFP) or Ad-45KD GRN virus for 24 h. Western blot analysis of RUNX2 and OPN in each groups. **D**, **E** Detection of mRNA and protein expression of PGRN in pVICs treated with NE for 24 h. **F** Detection of mRNA expression of PGRN in pVICs treated with NE or Alvelestat for 24 h under OM induction. **G-H** Western blot analysis of 45KD GRN in each groups. **I**–**K** Western blot analysis of PGRN and 45KD GRN in HEK293 cell treated with PGRN, NE, Alvelestat or both. *P < 0.05, **P < 0.01 vs corresponding control groups. NS meant no significant difference. The measurement data are expressed as the mean ± SD. All experiments were independently repeated three times

We previously reported that 45KD GRN, the degradation fragment of PGRN is markedly increased in the calcified AV tissues and we further studied the effect of full-length PGRN and 45KD GRN on valve calcification [4]. As shown in Fig. 6A, PGRN inhibited the expression of RUNX2 and OPN induced by OM, while 45KD GRN had the opposite biological function. PGRN can be cleaved into short peptides by NE [5]. Therefore, we hypothesized that the increase of 45KD GRN in calcified valves was due to elevated NE levels. We examined the effect of NE treatment on 45KD GRN in the presence or absence of calcification induction in pVICs. To evaluate whether NE can regulate PGRN at the transcriptional level, mRNA levels of PGRN were assessed by quantitative real-time PCR after treatment with NE in the presence or absence of OM. The Q-PCR results showed that there was no significant difference in mRNA level of PGRN after NE treatment (Fig. 6D, F). However, Western blot results showed that 45KD GRN, the degradation fragment of PGRN was notably increased after NE treatment (Fig. 6E, G). Similarly, in 293 cells, NE treatment also reduced the full-length PGRN and promoted the production of 45KD GRN (Fig. 6I).

### NE accelerates pVICs osteogenic responses through NF-κB and AKT.

**Fig. 7:**
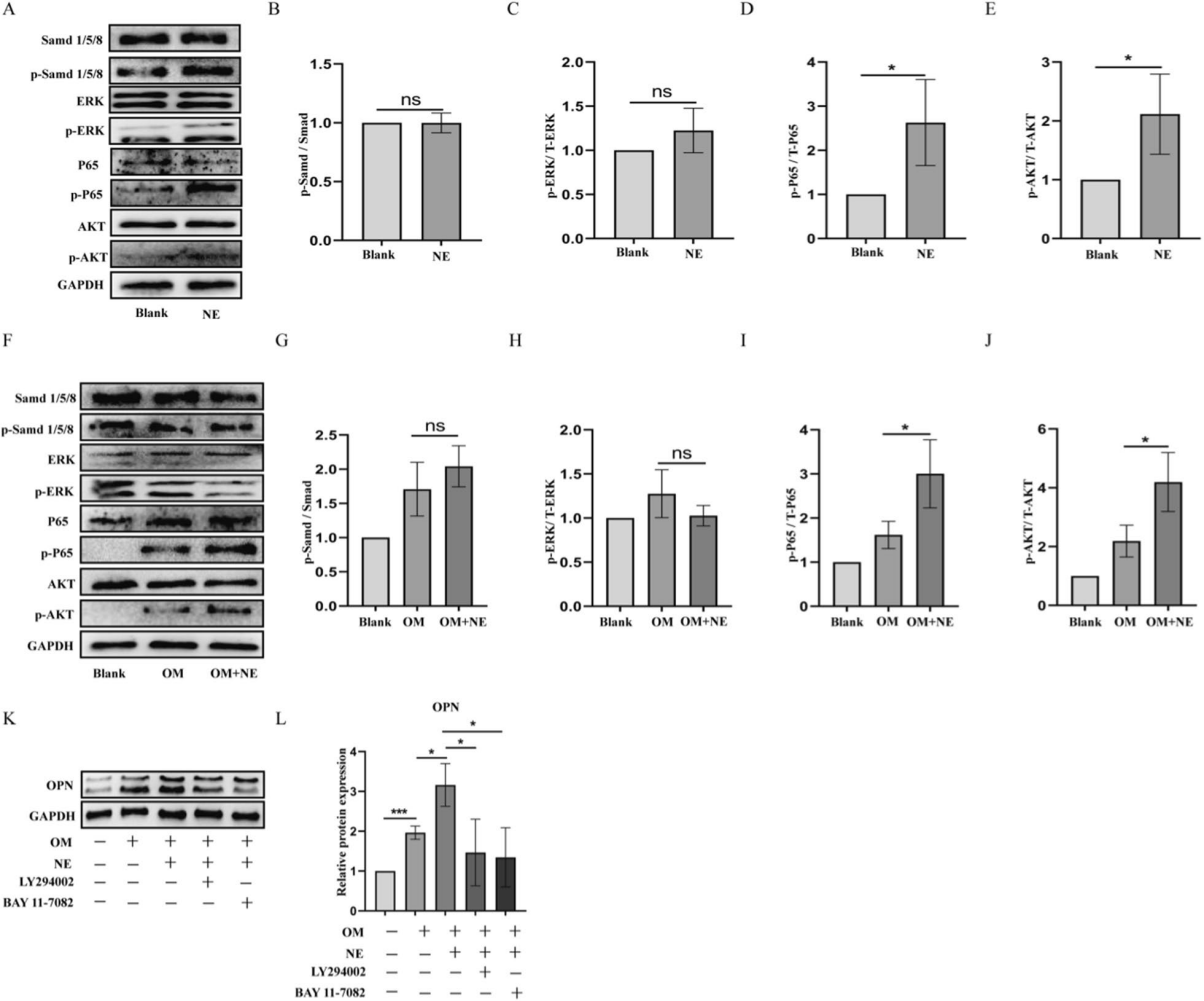
Intracellular signal pathways involved in NE effects. **A**–**E** pVICs were treated with NE for 24 h in normal medium or in osteogenic medium. Western blot analysis of Smad 1/5/8, phosphorylated Smad 1/5/8 (p-Smad 1/5/8), ERK, phosphorylated ERK (p-ERK), P65, phosphorylated P65 (p-P65), AKT and phosphorylated AKT(p-AKT). **F**–**J** Western blot analysis of Smad 1/5/8, p-Smad 1/5/8, ERK, p-ERK, P65, p-P65, AKT and p-AKT. **K**, **L** pVICs were treated with 10 nmol/L LY294002 and BAY11-7082 1 h before the addition of NE. The expression of OPN was measured by Western blot. All experiments were performed at least in triplicate. Data are presented as mean ± SD. *P < 0.05, **P < 0.01, ***P < 0.001 vs corresponding control groups

The possible intracellular mechanisms by which NE exerts its procalcification effects in pVICs were analyzed. We examined the effects of NE on classical osteogenic signaling pathway Smad1/5/8, non-classical osteogenic related signaling pathway ERK, AKT and NF-κB. As shown in Fig. 7A, NE induced phosphorylation of AKT and NF-κB while had no effect on phosphorylation of smad 1/5/8 and ERK. Furthermore, NE promoted OM-induced phosphorylation of AKT and NF-κB (Fig. 7F). LY294002, the inhibitor of AKT pathway and BAY11-7082, the inhibitor of NF-κB pathway, were used to evaluate whether they can reverse the pro-calcification effect of NE. LY294002 and BAY11-7082 were significantly blocked NE-induced upregulation of osteogenic markers OPN expression (Fig. 7K, L).

### Pharmacological inhibition of NE reduces valve thickness and fibrosis in APOE^−^/^−^ mice

**Fig. 8:**
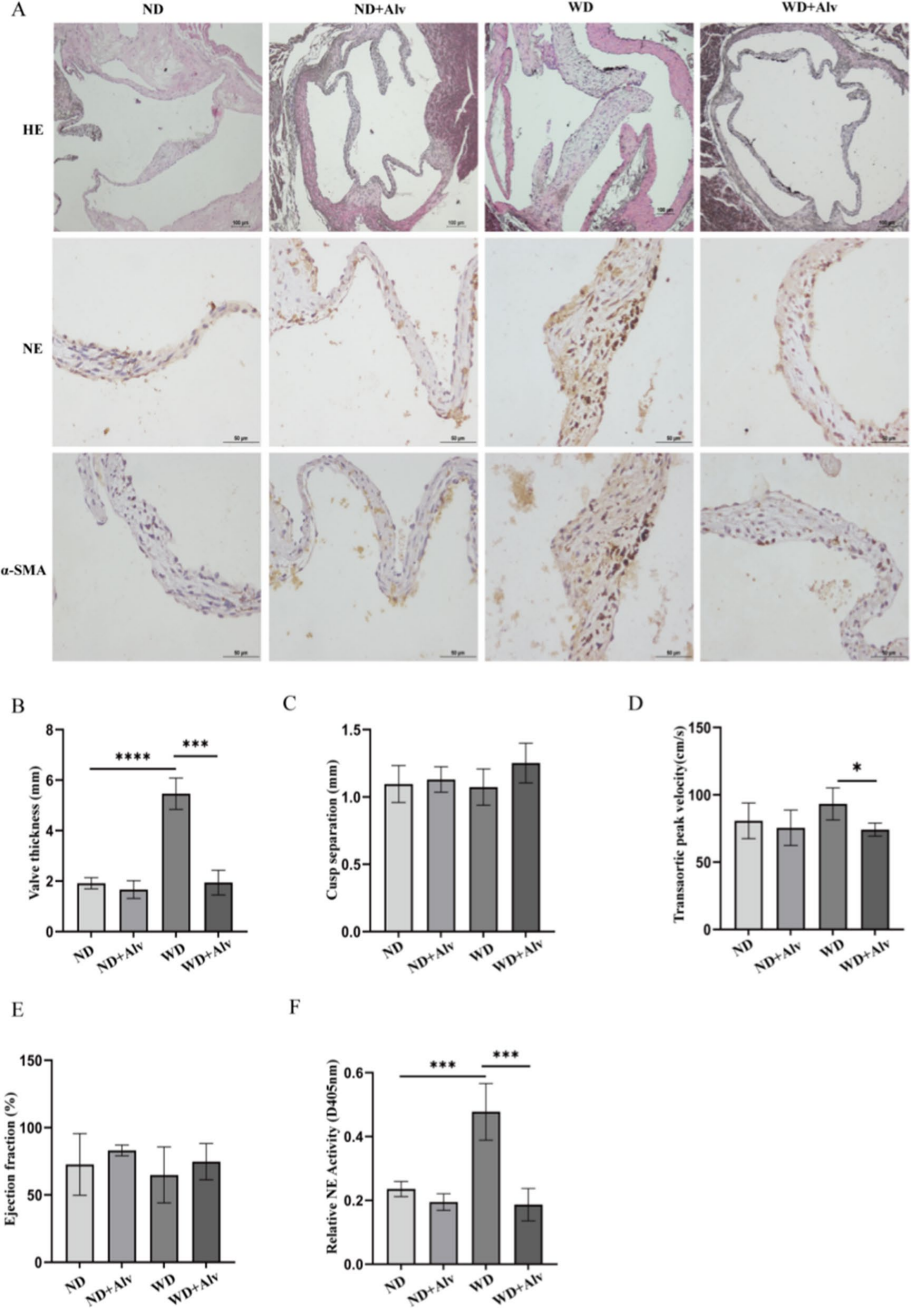
Alvelestat ameliorates valve thickness and partly improves cardiac functions of APOE^−^/^−^ mice. **A** HE and immunohistochemical staining analysis of NE and α-SMA expression of aortic valve in APOE^**−**^**/**^**−**^ mice. For normal diet group (ND), n = 5. For normal diet + Alvelestat group (ND + Alv), n = 4. For western diet group (WD), n = 3. For western diet + Alvelestat group (WD + Alv), n = 5. Scar bar = 100 µm, Scar bar = 50 µm. **B** Quantitative analysis of valve thickness in each group. **C**–**E** cusp separation, transaortic peak velocity and ejection fraction (EF) were measured by echocardiography in each group. **F** NE activity in each group was measured by N-methoxysuccinyl-Ala-Ala-pro-Val-p-nitroanilide. This experiment weas independently repeated three times. *P < 0.05, ***P < 0.001, ****P < 0.0001 vs corresponding control groups. The measurement data are expressed as the mean ± SD

To further explore the translational value of NE inhibition in prevention of valve calcification, Alvelestat was orally administered to APOE^**−**^**/**^**−**^ mice. As shown in Fig. 8A and B, APOE^**−**^**/**^**−**^ mice fed the western diet showed significant valve thickening and increased NE and fibrotic markers α-SMA. While Alvelestat significantly reduced the valve thickness and NE and α-SMA expression. We then used echocardiography to assess left heart function in APOE^**−**^**/**^**−**^ mice. WD group exhibited increased transaortic peak velocity compared with ND + Alv group, while other indicators, such as EF and cusp separation, were not statistically different (Fig. 8C–E). Meanwhile, increased NE activity was observed in WD group compared with ND group. Significantly lower levels of NE activity were observed in mice administered Alvelestat (Fig. 8F).

### Discussion

For decades, CAVD was considerd as a passive degenerative disease. But recent studies have shown that it is an active and dynamic disease which is mediated by endothelial dysfunction, inflammation and lipid deposition [16]. In terms of pathogenic factors, CAVD shares many pathophysiological features with atherosclerosis. Aortic valve tissue is divided into three layers: fibrosa, spongiosa and ventricularis [17]. During the disease progression, the valve leaflets gradually become thicker and stiff, with structural and collagen disorders [18]. In line with this, we found that compared with normal valve tissue, calcified valves were significantly thickened with increased elastin fiber fragments and calcium nodules. The valve interstitial cells (VICs) are the main cell type of valve. VICs exhibits mesenchymal phenotype characterised by high expression of α-SMA (alpha-smooth muscle actin) and vimentin instead of CD31 [19]. Therefore, we used these mesenchymal markers to phenotypic identify isolated porcine valve interstitial cells prior to the initiation of in vitro experiments (Additional file 1: Fig. S1).

Inflammation is essential for initiating valve calcification and accompanying the entire process of calcification [20]. Meanwhile, NE is closely associated with some inflammatory diseases such as arthritis [21] and acute pancreatitis [22]. NE levels are rapidly upregulated during inflammatory responses. The first finding of this study was that the expression and activity of NE of CAVD-patients was higher than that of control group. Besides, In vitro osteogenic medium also promoted the expression and activity of NE in pVICs, suggesting that it may play an important role in valve calcification. This experiment confirmed for the first time that valve interstitial cells could also secrete NE. In fact, NE was not only produced by neutrophils, but also secreted by macrophages and endothelial cells [23]. Since previous studies have reported that there are infiltration of macrophages, monocytes and other inflammatory cells in the pathological valve tissue [24], we consider that these inflammatory cells may be one of the sources of NE in calcified valve tissues. Our team will further investigate the mechanism of NE elevation in calcified valves.

Apoptosis is a crucial regulator of initiation and progression of CAVD [25]. Meanwhile, it is reported that NE could induce endothelial cells apoptosis [26]. Here, we found that NE promoted bax protein level with or without osteogenic medium induction. NE had no significant effect on the expression of bcl-2 under the conditions of OM induction. We speculate that it may be due to the negative feedback regulation mechanism of pVICs activated by OM. In general, NE exhibited the effect of promoting pVICs apoptosis, which suggests that NE may partly influence the osteogenic differentiation of pVICs through apoptosis.

In healthy valves, pVICs remain in a quiescent state [27]. With the change of valve homeostasis, pVICs are activated and further differentiated into myofibroblast-VIC characteristiced by high expression of α-SMA and osteogenic-VIC characteristiced by high expression of RUNX2 [28]. Therefore, we examined the effect of NE treatment on VIC phenotypic transformation at different time points. Our results demonstrated that NE promoted the activation of pVICs in the early stage, and then promoted the osteogenic differentiation of pVICs accompanied by an increase in inflammatory factors.

We then evaluated the role of NE on VIC during OM induction. As expected, NE aggravated the osteogenic differentiation, apoptosis and infalmmation of pVICs induced by OM, together with increased ALP and calcium deposits. However, these results were reversed by NE inhibitor Alvelestat. Alvelestat (AZD9668) is a novel, orally bioavailable NE inhibitor [29]. Early NE inhibitors, such as Sivelestat, bind irreversibly to NE, which causes serious side effects [30]. Compared to Sivelestat, the interaction between Alvelestat and NE is rapidly reversible. Moreover, clinical studies were conducted on pharmacology, tolerability and safety of Alvelestat. The results showed that Alvelestat could be quickly absorbed after oral administration, eliminating half-life between 5 and 15 h, and most of Alvelestat could be eliminated by the kidneys [31]. Therefore, it is less toxic than other early NE inhibtors. At present, the application of Alvelestat in the treatment of COPD and bronchiectasis has entered the clinical phase II study, suggesting its better clinical application prospect [32–34]. Alvelestat also has been reported to be protective in some inflammatory diseases, such as abdominal aortic aneurysms [35] and cystic fibrosis [36]. However, its role in cardiovascular diseases, especially CAVD, has not been studied. Hence, we further studied the role of Alvelestat in OM-induced osteogenic differentiation of pVICs. We chose a concentration of 40 nmol/L Alvelestat for this experiment, because previous studies has reported that the pIC50 values of Alvelestat for the whole-blood and cell-associated assays was about 40 nmol/L [33]. Our analysis indicated that Alvelestat could effectively inhibit the increase in NE activity and expression induced by OM. Meanwhile, Alvelestat also had a certain inhibitory effect on apoptosis and inflammation. These results suggest the potential of Alvelestat to inhibit osteogenic differentiation of pVICs. To achieve satisfactory inhibition of NE, we next used siRNA against NE to knockdown the NE mRNA expression during OM induction. Similarly, NE silencing also reduced OM-induced apoptosis, inflammation and osteogenic differentiation of pVICs. In addition, in order to further increase the clinical value of Alvelestat, we then explored the effect of Alvelestat on APOE^−^/^−^ mice fed with western diet. Our results showed that Alvelestat markly remitted the thickening and fibrosis of the valves. The results of echocardiography were further revealed that Alvelestat could decreased the transaortic peak velocity to alleviate cardiac function in APOE^−^/^−^ mice fed with western diet. Although there was no statistical difference in trends, mice in the WD group showed decline in cusp separation and ejection fraction. The reason for this phenomenon may be due to the small number of mice in the WD group. Therefore, the number of mice in WD group needs to be expanded to obtain more stable results.

Our group previously investigated the role of the inflammatory regulator PGRN in valve calcification and we found for the first time that 45KD GRN, a degraded fragment of PGRN, had a characteristic increase in calcified valve tissues. We demonstrated that full-length PGRN antagonized TNF-α and inhibited valve calcification while 45KD GRN played the opposite biological function of pro-inflammatory and pro-calcification [12]. So why does the degradation of PGRN to 45KD GRN increase when the valve is calcified? We hypothesized that this phenomenon may be caused by the elevation of a certain enzyme related to PGRN degradation. It is well known that PGRN can be cleaved by neutrophil elastase and degraded into short peptides called GRN [13]. Our experimental results showed that NE levels and activity were significantly increased during valve calcification. In vitro experiments showed that NE promoted the production of 45KD GRN in the presence or absence of OM. Overall, NE plays its role in promoting valve calcification at least partially by degrading PGRN to 45KD GRN.

We then explored the signaling pathways involved in NE promoting osteogenic differentiation in pVICs. The effect of NE seems to be mediated by several pathways. It has been reported that NE increased the nuclear translocation of NF-κB p-P65 and thus relieved LPS-induced lung injury of rats [37]. Another research reported that NE activated ERK and induced IL-8 production [38]. In addition, NE also induced tumor cell survival and migration via Src/PI3K-dependent activation of AKT signaling [39]. However, the effect of NE on Smad 1/5/8 signaling pathway is unclear. In our current study, we found that NE did not affect the classical osteogenesis Smad 1/5/8 signaling pathway and the non-classical osteogenesis-related ERK signaling pathway, but activated the NF-κB and AKT signaling pathway in the presence or absence of OM. Further verification was carried out by using BAY11-7082, the inhibitor of NF-KB, and LY294002, the inhibitor of AKT pathway.

### Conclusion

We have discovered that NE accelerates osteogenic differentiation of pVICs through promoting the production of 45KD GRN and activating the NF-KB and AKT pathways, thus enhancing valve calcification progression. In addition, we found that inhibition of NE by Alvelestat significantly attenuates valve calcification induced by OM. These findings open the possibility that NE may be a potential therapeutic targets for CAVD.

## Supplementary Information

**Additional file 1: Table S1.** Basic characteristics of control individuals and patients with CAVD. **Table S2.** The sequence of primers for Qpcr. **Table S3.** The information of antibodies used for western blot, immunohistochemistry, and Immunofluorescence. **Figure S1.** Phenotype identification of primary porcine aortic valve interstitial cells. Representative images of immunofluorescence staining of CD31, α-SMA and Vimentin in porcine aortic valve interstitial cells. Scale bar=50 μm. **Figure S2.** Identification of absorbed NE in porcine aortic valve interstitial cells. pVICs were treated with NE (1 µg/ml) for 4 h. Immunofluorescence staining was used to detect the Intracellular NE fluorescence intensity. Scale bar=25 μm.

## Data Availability

The data used and/or analysis during the current study are available from the corresponding author on reasonable request.

## Abbreviations

CAVD: Calcific aortic valve disease
CAVD: Calcific aortic valve stenosis
NE: Neutrophil elastase
pVICs: Porcine aortic valve interstitial cells
WB: Western blot
IHC: Immunochemistry
RT-PCR: Reverse transcription polymerase chain reaction
AV: Aortic valve
OM: Osteogenic medium
Runx2: Runt-related transcription factor 2
OPN: Osteopontin
HE: Hematoxylin and eosin
ARS: Alizarin red staining
bcl-2: B-cell lymphoma 2
bax: Bcl-2-associated x protein
α-SMA: Alpha-smooth muscle actin
TNF-α: Tumor necrosis factor alpha
IL-6: Interleukin-6
IL-1β: Interleukin 1 beta
ERK: Extracellular regulated protein kinases
NFκB: Nuclear factor kappa B
EF: Ejection fraction
