# Supplementary material for: The role of neutrophil elastase in aortic valve calcification
Source: J Transl Med. 2022 Apr 9;20:167. doi: 10.1186/s12967-022-03363-1 (PMC8994374; doi:10.1186/s12967-022-03363-1)
Supplement: Supplementary file 1 — Additional file 1: Table S1. Basic characteristics of control individuals and patients with CAVD. Table S2. The sequence of primers for Qpcr. Table S3. The information of antibodies used for western blot, immunohistochemistry, and Immunofluorescence. Figure S1. Phenotype identification of primary porcine aortic valve interstitial cells. Representative images of immunofluorescence staining of CD31, α-SMA and Vimentin in porcine aortic valve interstitial cells. Scale bar=50 μm. Figure S2. Identification of absorbed NE in porcine aortic valve interstitial cells. pVICs were treated with NE (1 µg/ml) for 4 h. Immunofluorescence staining was used to detect the Intracellular NE fluorescence intensity. Scale bar=25 μm. [file 12967_2022_3363_MOESM1_ESM.docx]

**Table S1.** Basic characteristics of control individuals and patients with CAVD

|  | Healthy control | Patients with CAVD |
| --- | --- | --- |
| n | 30 | 58 |
| Age, year | 60.7±6.1 | 57.1±12.1 |
| Male | 15（50％） | 30（51.7％） |
| Hypertension | 0 | 7（12％） |
| Diabetes mellitus | 0 | 2（3.4％） |
| β blockers | 0 | 14（24.1％） |
| ACE inhibitor | 0 | 9（15.5％） |
| Statins | 0 | 3（5.1％） |
| Angiotensin receptor blockers | 0 | 5（8.6％） |
| Diuretics | 0 | 34（58.6％） |
| Hyperlipidemia | 0 | 2（3.4％） |
| Arteriosclerosis | 0 | 11（18.9％） |
| Other CV disease | 0 | 10（17.2％） |
| LVEF(％) | - | 57.4±10.73 |
| FS(％) | - | 30.8±6.11 |
| SV(ml/B) | - | 72.3±25.78 |

Data are expressed as mean±SEM. ACE indicates angiotensin-converting enzyme; CAD, coronary artery disease; CV, coronary artery; LVEF, left ventricular ejection fraction; FS, fractional shortening; SV, Stroke Volume.

**Table S2.** The sequence of primers for qPCR

| Gene name | Primer sequences(5′ → 3′) | Species |
| --- | --- | --- |
| GRN | F:ATGGTCAGTTCTGCCCTGTG  R:GTGTTGTGGGCCATTTGTCC | Human |
| GAPDH | F:TCCAAAATCAAGTGGGGCGA  R:AAATGAGCCCCAGCCTTCTC | Human |
| GRN | F:GTCGGCAAGGGACACTGTAA  R:TGTTCAGTCCGGTCACCATG | Sus scrofa |
| RUNX2 | F:CCGAGACCAACAGAGGCATT  R:TCACTGTGCTGAAGAGGCTG | Sus scrofa |
| OPN | F:CGACGAGTCTCATCACTCCG  R:GGAAATCGGTGACCAGCTCA | Sus scrofa |
| α-SMA | F:CTGGACGTACGACTGGCATT  R:GTGGGTGACACCATCTCCAG | Sus scrofa |
| Collagen I | F:AAGATGTGCCACTCCGACTG  R:AGACTTTGATGGCGTCCAGG | Sus scrofa |
| TNF-α | F:CTGTGCCTCAGCCTCTTCTC  R:AACCTCGAAGTGCAGTAGGC | Sus scrofa |
| IL-6 | F:GCAGTCACAGAACGAGTGGA  R:CTCAGGCTGAACTGCAGGAA | Sus scrofa |
| IL-1β | F:GCTGATGGCCCCAAAGAGAT  R:AGAGCTGGTGGGAGATTTGC | Sus scrofa |
| GAPDH | F:TGGTGTCTTCACGACCATGG  R:GGTTCACGCCCATCACAAAC | Sus scrofa |

**Table S3.** The information of antibodies used for western blot, immunohistochemistry, and Immunofluorescence.

| Antibodies | Dilution | Catalogue | Application |
| --- | --- | --- | --- |
| PGRN | 1:1000 | R&D, AF2420 | WB |
| GAPDH | 1:2000 | Proteintech, #60004-1-lg | WB |
| Vimentin | 1:50 | Wanlei，WL01960 | IF |
| CD31 | 1:100 | Cell Signal Technology, #92841 | IF |
| RUNX2 | 1:500 | Santa, sc-390351 | WB |
| OPN | 1:500; 1:100 | Wanlei, WL00691 | WB; IHC |
| α-SMA | 1:1000; 1:50 | Abcam, ab5694 | WB; IF |
| Collagen I | 1:500 | Wanlei, WL0088 | WB |
| TNF-α | 1:500 | Wanlei, WL01581 | WB |
| bax | 1:500 | Wanlei, WL01637 | WB |
| bcl-2 | 1:500 | Wanlei, WL01556 | WB |
| NE | 1:500; 1:100; 1:50 | Bioss, bs-23548R | WB;IHC;IF |
| Smad 1/5/8 | 1:1000 | Cell Signal Technology, #12656 | WB |
| p-Smad 1/5/8 | 1:1000 | Cell Signal Technology, #13820 | WB |
| ERK1/2 | 1:1000 | Cell Signal Technology, #4695 | WB |
| p-ERK1/2 | 1:1000 | Cell Signal Technology, #4370 | WB |
| P65 | 1:1000 | Cell Signal Technology, #8242 | WB |
| p-P65 | 1:1000 | Cell Signal Technology, #3033 | WB |
| AKT | 1:1000 | Cell Signal Technology, #4691 | WB |
| p-AKT | 1:1000 | Cell Signal Technology, #4060 | WB |


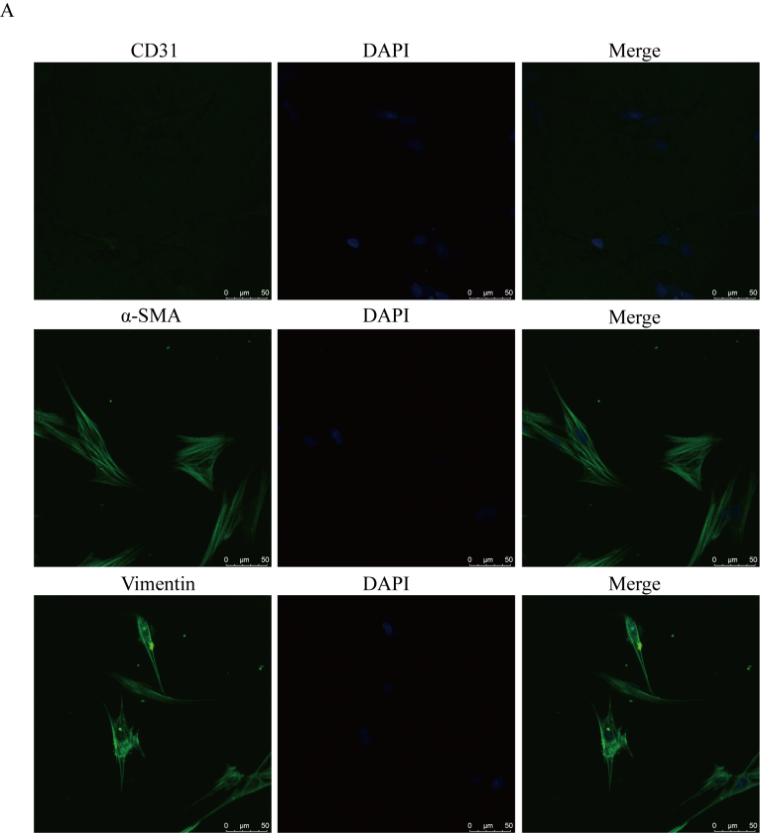


**Figure S1. Phenotype** **identification of primary** **porcine aortic valve interstitial cells.** Representative images of immunofluorescence staining of CD31, α-SMA and Vimentin in porcine aortic valve interstitial cells. Scale bar=50 μm.


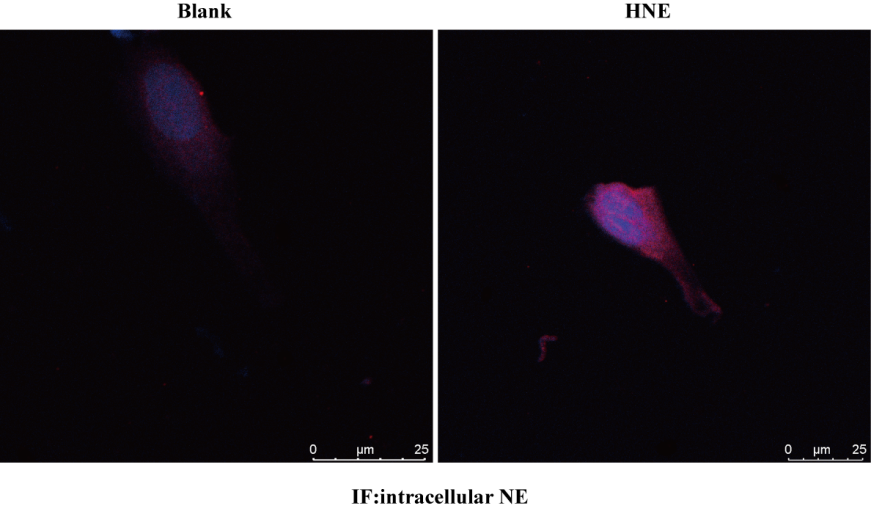


**Figure S2. Identification of absorbed NE in porcine aortic valve interstitial cells.** pVICs were treated with NE (1ug/ml) for 4 h. Immunofluorescence staining was used to detect the Intracellular NE fluorescence intensity. Scale bar=25 μm.
